# Can mental fatigue affect perception of barbell velocity in resistance training?

**DOI:** 10.1002/ejsc.12105

**Published:** 2024-04-23

**Authors:** Ruggero Romagnoli, Luca Filipas, Maria Francesca Piacentini

**Affiliations:** ^1^ Department of Human Movement and Health Sciences University of Rome “Foro Italico” Rome Italy; ^2^ Italian Weightlifting Federation ‘FIPE’ Rome Italy; ^3^ Department of Biomedical Sciences for Health Università degli Studi di Milano Milan Italy; ^4^ Department of Endocrinology, Nutrition and Metabolic Diseases IRCCS MultiMedica Milan Italy

**Keywords:** autoregulation training, cognitive fatigue, mental exertion, motivation, perceived velocity scale, velocity‐based training

## Abstract

Perception of Velocity (PV) is the ability to estimate single repetition velocity during resistance training (RT) exercises. The main purpose of the study was to evaluate the effects of Mental Fatigue (MF) on the accuracy of barbell PV. The secondary aims were to evaluate whether MF affected RT performance and ratings of perceived exertion (RPE; OMNI‐RES) in the back squat. Twenty‐four (14 Females, 10 Males) resistance‐trained participants underwent 2 familiarization sessions and 1RM test for the back squat. In two separate sessions, PV was tested for light, medium, and heavy loads in 2 conditions in random order: at rest (REST) and in MF condition (POST‐MF) induced by previous incongruent Stroop color‐word task. MF and Motivation were assessed through visual analog scales (VAS; 0–100) before and after the Stroop task. For each load subjects performed 2 repetitions and reported the RPE value. Mean propulsive velocity (Vr) of the barbell was recorded with a linear encoder, while the perceived velocity (Vp) of the subjects was self‐reported using the Squat‐PV scale. The PV accuracy was calculated through the delta score (ds: Vp–Vr). Following the Stroop task MF increased significantly (*p* < 0.001; *F* (1, 23) = 52.572), while motivation decreased (*p* < 0.05; *F* (1, 23) = 7.401). Ds, Vr, and RPE did not show significant differences between conditions (*p* > 0.05) for the three loads analyzed. MF induced by previous demanding cognitive task did not affect PV accuracy. Furthermore, subjects maintained unchanged both RT performance and RPE values associated with each load, even when mentally fatigued.

## Highlights

- Prior demanding cognitive task such as 45‐min of incongruent Stroop color‐word task induces MF.
- The perception of barbell velocity is a stable subjective parameter that remains unchanged even if the practitioners are mentally fatigued.
- MF does not affect the ability to achieve target velocities and the RPE values experienced for a given load.

## 1 INTRODUCTION

Acute mental fatigue (MF) is a psychobiological state that may arise during or after prolonged demanding cognitive activity and it is characterized by feelings of tiredness or even exhaustion and reduced ability to complete cognitive tasks (Habay et al., 2021; Van Cutsem et al., 2017). MF has implications for many aspects of daily life and has an adverse effect on cognitive and physical performance (Brown et al., 2020; Pageaux & Lepers, 2016). In recent years, interest in this latter aspect has grown and a substantial amount of literature is emerging in sports sciences to evaluate the effects of MF on different physical performances (Filipas et al., 2021; Marcora et al., 2009; Sun et al., 2021).

It has been well documented that MF impairs endurance performance through an increase in the rating of perceived exertion (RPE) (Filipas, 2021; Habay et al., 2023; Marcora et al., 2009; Van Cutsem et al., 2017). Furthermore, a recent systematic review (Habay et al., 2021) highlighted that MF also impairs sport‐specific psychomotor performance in a variety of sports by negatively influencing accuracy and reaction time. On the other hand, maximal strength, power, and anaerobic work appear not to be negatively affected by MF (Van Cutsem et al., 2017). It was reported that maximum voluntary contractions (MCV) performance of the knee extensor, countermovement jump performance, and mean cycling power during Wingate anaerobic tests were not impaired by prior MF (Van Cutsem et al., 2017). These data indicate that force production is not impaired by previous involvement in cognitive tasks.

Different prolonged cognitive tasks are typically used in studies to induce MF, such as transcription tasks, Stroop color‐word tasks, the AX‐Continuous Performance Tests (AX‐CPT), smartphone use, or playing video games (Habay et al., 2021; Van Cutsem et al., 2017). Among studies, the most used interventions generally ranged from 30 to 100 min (Brown et al., 2020; Van Cutsem et al., 2017). Intervention‐induced MF is generally assessed through self‐reported subjective measures (such as visual analog scales [VAS]), behavioral manipulation check (short version of Stroop task or AX‐CPT) or neurophysiological parameters such as electroencephalography (EEG) signals (Brown et al., 2020; Van Cutsem et al., 2017).

Although many studies are focusing on the effects of MF on exercise performance, research on resistance training (RT) to date is limited. A recent systematic review and meta‐analysis explored the effects of MF on strength endurance including 7 studies (Alix‐Fages et al., 2023). The results show that MF, compared to the control condition, significantly reduced the number of repetitions performed in both upper and lower body exercises. Therefore training volume can be compromised due to MF.

However, to our knowledge, no studies have explored the effects of MF on resistance training performance by analyzing the barbell velocity (as an intensity index) over a different range of loads. Typically, RT intensity refers to the relative load, that is, the percentage of the one‐repetition maximum (1RM). It is also possible to use barbell velocity to determine the exercise intensity. This method, called velocity‐based training (VBT), based on the close relationship between load and velocity at which the load is moved, offers a number of advantages such as prescription, monitoring, and real‐time feedback of RT parameters, that is, intensity and volume (Marston et al., 2022). In addition to the possibility of predicting 1RM without having to perform maximal tests (Guerriero et al., 2018; Marston et al., 2022), it is possible to monitor exercise intensity in real time right from the early stages of the warm‐up through the use of electronic devices such as linear position transducers (LPT) or accelerometers/inertial sensors (Pérez‐Castilla et al., 2019; Rum et al., 2022). It is therefore a very accurate objective method to prescribe, monitor, and eventually make adjustments in RT sessions (Shattock & Tee, 2022).

Given the attention that VBT is receiving in sports science (Guerriero et al., 2018), a very recent line of research is investigating how athletes are able to discriminate barbell velocities during training (Bautista et al., 2014, 2016; Lazarus et al., 2021; Romagnoli & Piacentini, 2022).

Perception of velocity (PV) is the ability to estimate single repetition velocity during RT exercises (Romagnoli & Piacentini, 2022). It has been demonstrated that, following a period of specific training with the combined use of LPT and PV scales, the barbell velocity perceived by the subjects (Vp) was very close to the real velocity (Vr) (Bautista et al., 2014, 2016). Although this line of research is growing and specific PV scales are being validated (Romagnoli, Civitella, et al., 2022), it is still unclear how fatigue can affect perceived velocity. From the preliminary data (Romagnoli, Lista, et al., 2022), it has been shown that muscular fatigue impacts real velocity (slower) but not the PV. Therefore, the main objective of the present study was to understand to which extent MF could cause alterations in velocity perception. Moreover, a further aim was to investigate whether MF could affect barbell velocity in order to extend knowledge on the effects of MF in specific RT performance settings. We hypothesized that MF would affect Vr but not PV.

## 2 MATERIAL AND METHODS

### 2.1 Participants

Twenty‐four (14 females and 10 males) active resistance trained volunteers (age = 24.4 ± 3.6 years; body mass = 64.9 ± 12.2 kg; height = 1.69 ± 0.08 m; 1RM (back squat) = 110.6 ± 29.6 kg; 1RM/BW (back squat) = 1.70 ± 0.30) with no experience in VBT participated in this study. Participants were healthy, with at least 2 years of RT experience (2–‐4 sessions per week) and regularly performed back squat exercises. All subjects were recreational practitioners, none of them were competitive weightlifters, CrossFitters, or bodybuilders. All participants had a medical clearance to participate in physical activities and no muscle or bone injury in the previous 6 months. In order to guarantee safety and proper performance, all sessions were supervised by a Certified Strength and Conditioning Specialist (CSCS‐NSCA) and three spotters. The G*Power software (G*Power V 3.1.9.7 Franz Faul, Universität Kiel) was used to calculate the sample size. The a‐priori power analysis was performed using *α* = 0.05 with a power = 0.80.

Written informed consent was obtained by all participants before starting the study and after receiving detailed information regarding the procedures. The study was conducted according to the guidelines of the Declaration of Helsinki and approved by the Institutional Review Board (CAR 109/2021).

### 2.2 Study design

The experimental design of this study consisted of five sessions: two familiarization sessions, one session to determine 1RM and two sessions of PV assessment, one with MF and a control session. A randomized and crossover design was used for the PV assessment sessions.

Participants were instructed not to perform any strenuous physical activity 48 h prior to the sessions. The sessions were performed with the same equipment and procedures and conducted by the same researchers at approximately the same time of the day (±1 h), separated by 48–72 h from each other.

In each session, a general dynamic warm‐up that included mobility exercises, bodyweight exercises, and dynamic stretching was followed by a specific warm‐up that included sets with progressive loads.

### 2.3 Familiarization and 1RM test

The first two sessions were used to familiarize subjects with the mentally fatiguing task (Stroop task) and to instruct them on the procedures and technical execution to be used in the back squat exercise (Romagnoli & Piacentini, 2022). Subjects carried out a short training with the combined use of the PV scale and the LPT. Participants received visual and auditory feedback on the velocity of each repetition from the LPT during execution, and at the end of the set they displayed all the velocities performed on the PV scale. The third session consisted of a progressive 1RM test (Shattock & Tee, 2022). Also during this test, the LPT and PV scales were used in combination.

Subjects started the test with 5 repetitions and an initial load of 20 kg. Thereafter, the load increased and the repetitions decreased according to the recorded mean propulsive velocity (MPV). 1RM was considered the load that they could lift only once with a good technical execution. During the test, subjects were motivated to give their best effort by strong verbal encouragement.

### 2.4 Experimental sessions

Perception velocity accuracy was evaluated with a blinded load test in two separate days, 1 day in rest condition (REST) and 1 day in MF condition (POST‐MF). The procedures were the same as those used in a previous study (Romagnoli, Civitella, et al., 2022). The test consisted of 3 different loads, administered in random order using an online software (random.org), chosen according to the velocities reached during the 1RM test (light: MPV ≥1 m·s^−1^, medium: 0.6 ≥ MPV ≤0.8 m·s^−1^ and heavy: ≤0.4 m·s^−1^). Three‐minute rest intervals were provided between sets. Participants performed 2 repetitions for each load, during which the MPV of the barbell (real velocity, Vr) was recorded. At the end of the set they had to identify the fastest repetition of the two and, using the squat PV scale (Romagnoli, Civitella, et al., 2022), they had to estimate the velocity of each of the 2 repetitions (perceived velocity, Vp) and report the values of RPE using the OMNI‐RES scale (Robertson et al., 2003).

### 2.5 Mental fatigue, stroop task, and VAS scales 0–100 (MF, motivation)

In the MF condition, subjects performed 45 min of a computerized and modified version of the incongruent Stroop color‐word task. The Stroop task demands response inhibition and sustained attention (MacLeod & MacDonald, 2000) and has previously been shown to induce MF (Van Cutsem et al., 2017). Participants performed this cognitive task on a computer (screen size: 35.4 × 19.9 cm), whilst sitting comfortably in a quiet, dimly lit room. This Stroop task consists of four words (yellow, blue, green, and red) serially presented on the computer screen, displayed until the participant responded, followed by a 1.5 s rest interval. Participants were instructed to press one of four colored buttons on the keyboard (yellow, blue, green, and red), with the correct response being the button corresponding to the ink color (yellow, blue, green, and red) of the word presented on the screen. The word presented and its ink color was randomly selected by the computer* with only incongruent stimuli combinations. Twenty‐four practice attempts were allowed to ensure the participant fully understood the instructions. Participants were instructed to respond as quickly and accurately as possible. Visual feedback was given after each word in the form of correct or incorrect response, reaction time, and accuracy so far. Responses faster than 200 ms were excluded from the analysis as it is likely the participant responded before seeing the word (Martin et al., 2016). Responses over 2 s were recorded as lapses and removed from the analysis (MacLeod & MacDonald, 2000). Participants familiarized with the Stroop task for 5 min during the preliminary visit. The reaction time of the correct responses and accuracy were averaged for 3 blocks of 15 min. The control condition consisted of an easy cognitive task (watching a documentary video (Predators Bloodlines, National Geographic UK, 2020)) performed under the same conditions as the Stroop task. Participants were instructed to sit quietly for 10 min in the same isolated room.

Visual analog scales (VAS) were used to assess the perceptions of MF and motivation toward the upcoming tests. Subjects were asked to place a mark on a 100 cm line to answer the questions “How mentally fatigued do you feel now?” and “How motivated do you feel now?”. The VAS were anchored at 0 with “Not mentally fatigue” and 100 with “Very mentally fatigued”, and at 0 with “No motivation at all” and 100 with “Maximal motivation” for the mental fatigue and motivation scales, respectively. The mark was then measured in mm to provide the VAS score.

## 3 STATISTICAL ANALYSIS

Descriptive statistics were determined using standard procedures and data distribution was tested with Shapiro–Wilk test. Data are reported as mean ± standard deviation or median ± interquartile range, according to the distribution presented. The accuracy of the PV is represented by the delta score (ds) which is determined by the difference between perceived velocity (Vp) and real velocity (Vr). Two‐way (condition × time) repeated measures ANOVA were used to compare the PRE and POST‐MF conditions for both MF and motivation. One‐way analysis of variance with Tukey's multiple comparisons test was used to determine the effects of time for reaction time and accuracy during the Stroop task. Qualitative analysis on fastest repetition performed during the PV test was conducted using Pearson's Chi‐Square test. Paired *T*‐tests were performed to compare Vr values between the two conditions (REST and POST‐MF) for each of the three loads. Comparisons between the two conditions for the RPE and ds variables were carried out using Wilcoxon tests.

A significance level of *p* < 0.05 was adopted. All data were collected and organized with Microsoft Excel (Microsoft Office 16; Microsoft Corporation, 2018) and analyzed using the Statistical Package for the Social Science (SPSS), version 25.0 (SPSS Inc.).

## 4 RESULTS

Perceived MF (*p* < 0.001; *F* (1, 23) = 52.572) increased significantly and motivation (*p* < 0.05; *F* (1, 23) = 7.401) decreased after the mental fatiguing task (Table 1).

**TABLE 1:** Visual analog scale values in pre and post stroop task or control condition.

Condition	Stroop task				Control			
	MF		Motivation		MF		Motivation	
	Mean (SD)	CI (95%)	Mean (SD)	CI (95%)	Mean (SD)	CI (95%)	Mean (SD)	CI (95%)
PRE	27.78 (22.78)**	18.17	55.07 (25.68)*	44.22	28.28 (20.25)	19.72	55.05 (23.11)	45.29
		37.40		65.90		36.83		64.81
POST	60.61 (25.41)**	49.88	45.99 (26.24)*	34.90	27.79 (19.20)	19.69	56.21 (23.25)	46.39
		71.34		57.07		35.90		66.02

Abbreviation: MF, Mental Fatigue.

*p < 0.05; **p < 0.001.

Reaction time during the Stroop task increased significantly during the task (*p* < 0.001; *F* (1.536, 35.32) = 81.76). Post‐hoc comparisons indicated differences between the first and the second block (*p* = 0.005), the first and the third block (*p* < 0.001), and the second and the third block (*p* < 0.001). No significant differences on accuracy were found (*p* = 0.160; *F* (1.612, 37.08) = 1.977).

MF did not impair the subjects' ability to identify the fastest repetition. In the blinded load tests, in REST condition subjects correctly identified the fastest repetition, that is, 77.8% of the time and in the POST‐MF condition, 80.6% of the time (*p* = 0.682).

**FIGURE 1:**
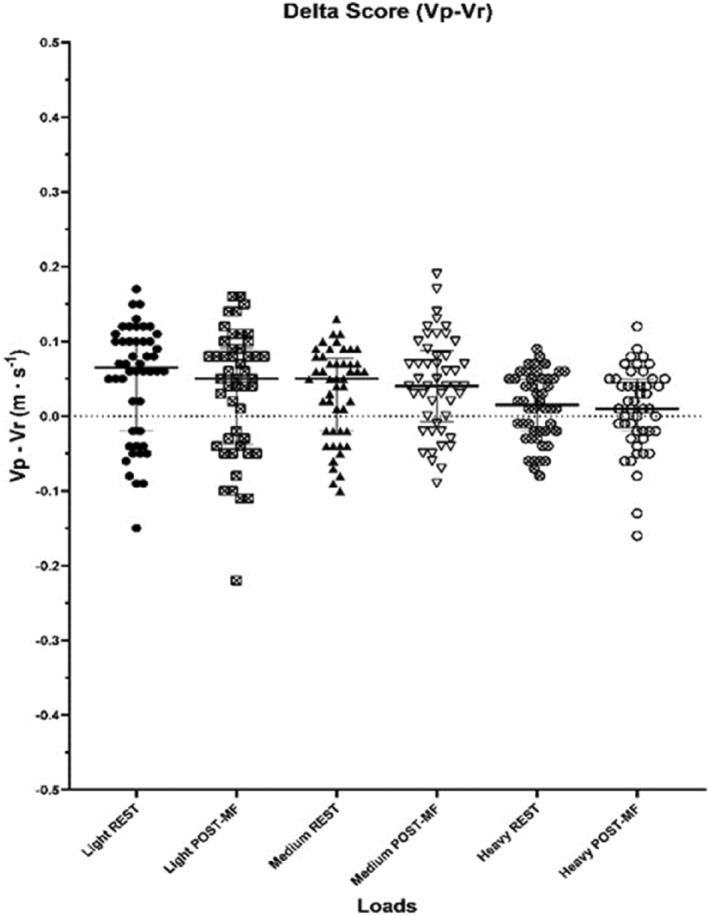
Delta Score (ds) in m·s^−1^ for the three loads.

Real velocity (Vr) remained unchanged at all loads (light loads: *p* = 0.195, medium loads: *p* = 0.058, heavy loads: *p* = 0.209) (Table 2). Similarly, ds and RPE were not significantly different between REST and POST‐MF conditions for the three loads considered (Figure 1 and Table 3) (light loads: *p* = 0.606 medium loads: *p* = 0.106, heavy loads: *p* = 0.912).

**TABLE 2:** Real velocity in m·s^−1^ for the three loads in REST and POST‐MF condition.

Condition	Light load		Medium load		Heavy load	
	Mean (SD)	CI (95%)	Mean (SD)	CI (95%)	Mean (SD)	CI (95%)
REST	0.98	0.95	0.60	0.57	0.37	0.35
	(0.08)	1.00	(0.07)	0.62	(0.07)	0.38
POST‐MF	0.97	0.94	0.58	0.56	0.36	0.34
	(0.08)	0.99	(0.07)	0.60	(0.07)	0.38

*Note*: Mean (± standard deviation); CI 95% = mean confidence interval (lower–upper limit).

**TABLE 3:** RPE values on the OMNI‐RES scale (0–10) shown as median (± interquartile range).

	Light load	*p*‐value	Medium load	*p*‐value	Heavy load	*p*‐value
REST	1.00 (1.75)	0.125	6.25 (2.50)	0.064	8.75 (1.38)	0.545
POST‐MF	1.50 (1.00)		6.00 (1.88)		8.50 (1.38)	

## 5 DISCUSSION

The aim of the present study was to investigate for the first time the effects of MF on the accuracy of the perception of barbell velocity, and to broaden the current knowledge on the effects of MF on RT performance. Overall, the results showed that MF induced by a 45‐min cognitive task did not compromise either the accuracy of barbell velocity perception or RT performance (Figure 1 and Table 2) despite a significant increase in MF (Table 1).

During the blinded load tests, subjects were asked to randomly lift light, medium, and heavy loads and for each load they had to perform two repetitions. To understand the participants' ability to discriminate the fastest of the 2 repetitions for each load, they were asked to indicate which of the two repetitions was the fastest. It has to be pointed out that often the two repetitions had a very similar velocity such as 0.66 and 0.65 m·s^−1^. If the subject indicated that the repetitions had the same velocity it was counted as an error, even if the difference was minimal. Our results showed no differences in the subjects' ability to discriminate the fastest repetition between the two conditions. It has previously been shown that this ability can be improved through longer familiarization periods in which subjects perform training sessions with the combined use of electronic devices and the PV scale, thus being more exposed to repetition velocity feedback (Romagnoli & Piacentini, 2022).

The delta score (i.e., difference between the velocity perceived (Vp) by the subject and the velocity (Vr) recorded through a linear position transducer) was used to investigate the effects of MF on PV accuracy. The closer the ds is to zero, the greater the individual's ability to predict the movement velocity of the barbell. Contrary to our hypothesis, MF did not affect ds of the three loads (Figure 1), thus revealing that the perception of velocity is not affected under MF conditions with this experimental setup. The study of perceived velocity during RT is an emerging novelty. While the results are encouraging, current knowledge is limited. Bautista et al. (2014) were the first to investigate the existence of this subjective parameter. Further studies confirmed that training with feedback on the execution velocity improves the accuracy in determining barbell velocity (Romagnoli & Piacentini, 2022). This result is comforting because it allows individuals to train in the correct velocity zone even in the absence of electronic devices. Thus, the results of this study provide further insight into PV by showing that, although it is a perceptual parameter, there are no variations in its accuracy following high cognitive demanding activities that induce MF. These results confirm that PV is a stable parameter even in more ecological settings and not just in laboratory test contexts.

A further aim of this study was to investigate the effects of MF on RT performance, which remains an underexplored topic despite the growing interest on the topic. Studies conducted so far indicate that MF has a clear negative effect on endurance performance (Pageaux & Lepers, 2016), especially isolation tasks in comparison to whole‐body endurance tasks (Giboin & Wolff, 2019). It has also been demonstrated that MF impairs sport‐specific psychomotor performance which is the cognitive processing of sensory and perceptual information in a sport‐specific context that results in highly complex motor behavior (Habay et al., 2021). MF negatively affects technical and decision‐making skills, reaction times, and accuracy, thus impairing performance as these are fundamental elements for success in a variety of sports (Filipas et al., 2021; Habay et al., 2021; Sun et al., 2021). In resistance training context, MF has been mainly explored by analyzing the number of repetitions performed for a given load (endurance strength). A recent systematic review and meta‐analysis (Alix‐Fages et al., 2023) analyzed data from 7 studies reporting that participants performed fewer repetitions to failure at a given relative load when they were mentally fatigued compared to the control condition, in both upper and lower body exercises. Therefore, before RT session it is recommended to avoid demanding cognitive tasks that can induce MF, as this negatively affects the total training volume (i.e., the number of repetitions × sets × load), which represents one of the most important parameters in resistance exercise prescription along with intensity (Schoenfeld et al., 2017). There is almost no information in the literature on the effects of MF on RT performance analyzing the intensity parameter that can be quantified or as percentage of the 1RM or through the velocity of the barbell, as demonstrated by the velocity‐based training method (Guerriero et al., 2018; Pareja‐Blanco et al., 2020). MF did not influence the exercise intensity and the subjects, even if mentally fatigued, were able to reach their target velocity and thus were able to express the same level of performance. No differences in Vr between the REST and POST‐MF conditions for the 3 loads analyzed (light, medium and heavy) were found. A recent study (Fortes et al., 2021) demonstrated that MF did not influence barbell velocity at 40% of 1‐RM, countermovement jump, and 100‐m and 200‐m dash performance. These results are in line with previous research (Brown et al., 2020; Van Cutsem et al., 2017) that demonstrated no effect of MF on short‐term maximal anaerobic performance.

A further result of the present study is that RPE was not affected by MF (Table 3). This is an interesting result as it is in contrast with what has been widely demonstrated in endurance performance, where MF induces a decline in performance and an increase in RPE (Marcora et al., 2009; Pageaux & Lepers, 2016). For other sports, the results are conflicting because not all studies on MF analyzed RPE values and when analyzed, some reported no differences and others reported an increase (Habay et al., 2021; Sun et al., 2021). Regarding resistance training, there is a lack of studies evaluating the effects of MF on the intensity and the corresponding RPE values. However, no differences in reported RPE values were found in most of the studies that investigated isometric maximal performance (Brown et al., 2020). Several authors found similar RPE values between control and MF conditions but a reduction in the maximum number of repetitions performed when subjects were mentally fatigued (Alix‐Fages et al., 2023). Thus showing that in MF conditions, the same RPE value is reached by performing fewer repetitions and therefore a reduced training volume.

Despite the novelty of the present study we have to acknowledge the limitations. Our sample consisted of participants with prior RT experience; therefore, the results may be specific to this population and not applicable to novice or unexperienced subjects. Further investigations should include subjects with different levels of experience in order to investigate the effects of expertise on PV. Although, as already specified, self‐reported subjective measures are valid tools to evaluate MF, no neurophysiological measurements (e.g., EEG signals) were used. We focused on 3 different loads for a single exercise; however, future studies should consider including more exercises simulating an entire RT session to provide additional interesting information. The choices for this experimental setup were made to make the protocol as similar as possible to a real training situation in the weight room.

## 6 CONCLUSION

In summary, this is the first study that has evaluated the effects of MF on the accuracy of PV and on RT performance in terms of intensity assessed via barbell velocity.

The results of this study demonstrated that even if mentally fatigued, the subjects were able to reach the same target velocities (Vr) experiencing the same RPE levels. Therefore, although cognitive tasks may induce MF, barbell velocity perception and RT performance were not affected.

## CONFLICT OF INTEREST STATEMENT

The authors report no conflict of interest.
